# More Time, Carrot-and-Stick, or Piling Coffins? Estimating the Role of Factors Overcoming COVID-19 Vaccine Hesitancy in Poland and Lithuania in the Years 2021–2022

**DOI:** 10.3390/vaccines10091523

**Published:** 2022-09-14

**Authors:** Marcin Piotr Walkowiak, Justyna B. Walkowiak, Dariusz Walkowiak

**Affiliations:** 1 Department of Preventive Medicine, Poznan University of Medical Sciences, 60-781 Poznań, Poland; 2 Department of Language Policy and Minority Studies, Adam Mickiewicz University in Poznan, 61-712 Poznań, Poland; 3 Department of Organization and Management in Health Care, Poznan University of Medical Sciences, 60-356 Poznań, Poland

**Keywords:** COVID-19, vaccination, vaccination coverage, trust in vaccine, interventions to increase vaccination coverage, public health

## Abstract

In this study, motivation for late (from 2021-W22, i.e., 24 July 2021) uptake of the first dose of the COVID-19 vaccine among adults in Poland and Lithuania is indirectly measured in order to avoid social-desirability bias or rationalisation in retrospect of prior decisions. Weekly vaccine uptake is modeled as if vaccine hesitant people were late adopters of a new product, with a fitted non-linear trend representing steadily decreasing interest. Before the analysed period, the vaccine uptake among Polish and Lithuanian adults was almost identical. Vaccination simply explainable by the trend was responsible for the vaccination of an additional 19.96% and 19.06% adults, respectively. The fear incurred by spikes in consecutive waves of infection motivated 3.20% and 3.89% more people, respectively, while the COVID-19 passport, introduced only in Lithuania, convinced an additional 13.98% of the overall population. The effect of the COVID passport was the biggest in the 18–24 age group, and the least visible among people aged 80 or more. In the latter group, other factors also had a limited impact, with merely 1.32% tempted by the one-time €100 payment offered to everybody aged 75 or more.

## 1. Introduction

Considering that the COVID-19 pandemic is a global threat, effective measures that would maximise vaccine uptake are of paramount importance for governments of any country. Not only do the healthcare authorities have to motivate citizens to get vaccinated but they must also cope with both antivaccine movements and those who consider pandemic government overreach a bigger threat than the virus. Particular countries embarked on that task in various ways and with varying success, employing measures ranging from emphasis on moral obligation, citizen duty, national solidarity (persuasion) via the mandatory option (legal obligation to get vaccinated) to various incentives, both negative (financial punishment for failure to vaccinate) and positive (financial reward for getting vaccinated) [1,2]. Similar to other countries, vaccine hesitancy is a problem in Poland and Lithuania, reported not only in terms of the population at large but even in the healthcare section [3].

Few measures are so punitive as having non-vaccinated citizens pay out of their own pocket for their coronavirus-related treatment—as was the case in Singapore [4]—or as making vaccination mandatory for all adults, the short-lived law (with fines of up to €3600 from mid-March for those who did not comply) introduced in Austria in February 2022 and repealed after just a month. However, in many countries worldwide COVID-19 passports were implemented that gave their owners more freedom in using public services and/or entailed restrictions for the remainder of the population [5], typically also leaving alternative options in the form of a recent COVID-19 test or a recently undergone coronavirus infection. COVID-19 certificates introduced in France in the summer of 2021, which enabled entry to cinemas, museums, cafés, trains, and other public venues, were reported to have increased vaccine uptake by the end of 2021 in that country by 13.0 (95% CI 9.7–14.9) percentage points (p.p.) of the total population. A similar certificate in Germany helped to increase vaccine uptake in that period by 6.2 (2.6–6.9) p.p. and in Italy—by 9.7 (5.4–12.3) p.p. [6], though the social and political cost borne by the decision-makers, such as massive protests e.g., in France or Austria, was considerable [7,8]. The effects of COVID-19 passports in Denmark, Israel, and Switzerland were also analysed by Mills and Rüttenauer [9], who found the results inconclusive in terms of the effectiveness of this measure. The effectiveness of COVID-19 passports in the UK has been questioned by de Figueiredo et al. [10].

A straightforward financial reward in return for getting vaccinated is far less frequently employed as incentive. To date, Serbia appears to be one of the few countries to implement that solution, albeit on a short-term basis, with its president having promised in May 2021 to pay an equivalent of approximately €25 to any citizen vaccinated by the end of that month [11]. In the North American state of West Virginia, members of the 16–35 age group were rewarded for vaccination with bonds worth $100 per person [11]. Some other randomly targeted rewards, financial or in kind, were introduced in other countries, such as state vaccine incentive lottery programmes in some American states [12], Canadian provinces [13], and Poland [14]. South Dum Dum Municipality in North Kolkata, India [15] introduced a 25% discount on pending property tax in return for full vaccination. Among the more imaginative incentives introduced in some US states, there were a few days’ free holiday stays in selected locations, free admission to amusement parks, complimentary drinks in restaurants, and even good time credit for prison inmates who decided to vaccinate [16]. Vouchers or bonuses have also been common.

Compared to other European states, the vaccine policy in Poland can be characterised as extremely lenient. Striving to avoid a drop in political support at all costs, the populist government repeatedly explicitly refused to coerce its citizens in any way to receive the vaccine [17,18,19,20,21]. Thus, a COVID-19 passport similar to that introduced in Lithuania, though recommended by many Polish healthcare professionals, was never implemented or even seriously considered at the governmental level of decision. There were some half-hearted attempts to encourage citizens, e.g., through a state-run incentive lottery, but despite considerable public money spent, the campaign was widely criticised as ineffective and its social resonance was meager [22]. At the same time the vaccine policy could be described as inconsistent both in terms of its content and the related information policy. For instance, the vaccine mandate for teachers and uniformed services, announced in December 2021 to take effect 1 March 2022 [5,23], was revoked in January 2022 for teachers [24] and shortly afterwards for the other professional group [25].

In contrast with Poland, the measures adopted in Lithuania combine the “stick” with the “carrot” element of the proverbial duo. The former is represented by the so-called “opportunity passport” (*galimybių pasas*), first introduced in that country concomitantly with COVID-19 vaccination as merely confirmation, with no benefits or restrictions entailed; somewhat later it came to be used to enable unrestricted entry to concerts and big cultural events. However, eventually a new law, introduced in August 2021 and in force since mid-September 2021, changed the game by effectively restricting access to hairdressers, beauty parlours, travel agents, workshops etc., as well as to supermarkets and larger shops for its non-passport holders [26].

Regarding the “carrot”, Lithuania was one of the few countries to offer a financial incentive in return for getting vaccinated. Discussed in the media for some time, the plan was finally officially announced at the beginning of October 2021 [27]. As admitted by the Minister for Social Security and Labour, Monika Navickienė, this payment was a desperate last resort, but she stressed that if it were to save more lives and protect more people and their loved ones, then that was the price the state would pay [28]. A reward of €100 was to be paid to people aged 75 or older, who between 1 September 2021 and 30 November 2021 were vaccinated according to the full vaccination schedule, or by 30 November 2021 received a booster dose of the vaccine. Those were to receive their money between 7 December 2021 and 23 December 2021, a well-timed move considering the cultural relevance of Christmas (and the tradition-related expenditure it entails) in Catholic Lithuania. The money was not to be subject to taxation, and its reception would not have any influence on other social benefits, such as compensation for heating costs [29].

### Study Purpose

The aim of this study is to determine which factors—and to what degree—vaccine hesitant adults in Poland and Lithuania were ultimately convinced to get vaccinated for COVID-19. The following potential factors come into play and are considered: restrictions associated with the COVID-19 passport (Lithuania), the financial incentive for people aged 75 or more (Lithuania), and the intimidating impact of consecutive peak waves (in both countries). While the third factor is a psychological phenomenon seemingly unrelated to active governmental efforts (though also to be reckoned with), the analysis of the first two factors is of special importance since it might offer practical feedback for policy-makers, including in other countries.

Contrary to other research, we decided to base our study directly on actual vaccination outcomes because relying on the declaration of intent is fraught with a risk of serious inaccuracy, as evidenced by the discrepancy observed even in a society as disciplined and collectively-minded as that of Japan: 10% of those declaring the willingness to vaccinate never actually did so [30]. In an American study, the corresponding percentage of those who had declared they would receive the vaccine and failed to do so was 8% [31]. Directly asking the respondents who did vaccinate about their motivation does not appear useful either, as they may feel the need to rationalise their decision in retrospect. Nevertheless, it is possible to analyse the people’s decisions indirectly, based on the actual vaccination data. Since Poland and Lithuania initially had almost identical overall adult vaccination rates, their comparison can be treated as a natural experiment. This is made easier also by the fact that both countries experienced peaks of the delta variant wave in different moments but also differed regarding policy: Lithuania adopted a COVID-19 passport and even subsequently resorted to paying €100 to each person aged 75 or older for getting vaccinated, whereas Poland did not implement any of these measures.

## 2. Materials and Methods

Data on the number of weekly vaccinations were taken from the ECDC website, as of 9 June 2022. For the purpose of the study, receiving a single dose of the COVID-19 vaccine is used as a metric of overcoming vaccine hesitancy. The analysed period is 2021-W22 to 2022-W21 (31 May 2021 to 29 May 2022).

For the purpose of the study, it is assumed that COVID-19 vaccine uptake follows general trends of adoption of a new product by consumers. While innovation adoption models tend to assume distribution akin to a bell curve, in contrast to goods whose adoption is initially limited by price or consumer awareness, initial vaccination was determined by the availability of doses [32,33,34,35]. However, in the middle of summer 2021, vaccine shortages ended, while the steadily waning interest among the remaining unvaccinated became the limiting factor.

Assuming the already existing factors, such as people slowly overcoming their vaccine hesitancy, bowing to peer pressure, or finally finding some time to book an appointment, each subsequent week there should be a lower number of those freshly vaccinated. This part of an idealised bell curve after inflexion point at one standard deviation after the mean could be approximated as a sum of shifted negative power functions.

In epidemiology there are techniques, such as mixed linear models, that are used to fit a function to a phenomena where a part of the observation unidirectionally diverges from the general trend [19]. They are especially useful for calculating the true toll of the pandemic based on the number of excess deaths instead of the number of detected cases. The same technique could be adapted to calculate the number of excess vaccination that diverges from innovation adoption model.

The waning interest of later adopters of vaccines could be roughly approximated as a negative power function. Theoretically, in order to fit a power function to a linear regression model, one needed to logarithm both sides of the equation:

$$y=ax^{b}\;ln\left(y\right)=bln\left(x\right)+ln\left(a\right)$$

However, applying directly a negative power function would mean assuming that the curve has an asymptote for *x* = 0, which is unlikely for any social phenomena. Additionally, as age groups had different vaccination starting points, one cannot assume that they would have the same shift. Thus, this model is calculated simultaneously for different possibilities, shifted by powers of 2, for *n* from 1 to 9.

The way of fitting is based on techniques derived from mixed linear models. Initially, simple regression model is fit. Subsequently, weights are calculated based on normalised residuals, though in the case of normalised residuals exceeding one, the weight is set to 0. Based on that, a weighted regression model is calculated and weights are recalculated and again trimmed for excess values before calculating final weighted regression model.

$$ln\left(y\right)=bln\left(x+2^{n}\right)+ln\left(a\right)$$

Fitting those functions would create a set of functions individually able to match empirical data. For an even better fit, in the next step all such functions are superimposed and fitted as potential explanatory variables in a new model.

$$y=d_{1}\left(a_{1}{\left(x+2^{1}\right)}^{b_{1}}\right)+d_{2}\left(a_{2}ln{\left(x+2^{2}\right)}^{b_{2}}\right)+\ldots +d_{9}\left(a_{9}ln{\left(x+2^{9}\right)}^{b_{9}}\right)+c$$

This crude model is highly likely to overfit the data. As *d* coefficient has to be non-negative, in the next step algorithm seeks for any negative *d* coefficient as a clear sign of overfitting. The *d* coefficient with the lowest value is eliminated, and the above-mentioned model is fitted once more. This procedure is repeated until all remaining *d* coefficients are positive.

With the final trend line, the observed weekly vaccinations are subdivided into those explainable by the trend and the excess above the trend. All observations are counted to be within the trend, unless for 4 weeks in a row they are above the trend. Additionally, in the case of Lithuania, an additional trend line was calculated only for the period 2021-W32 to match the new trend line from the introduction of the COVID-19 passport. As the base trendline value for Lithuania, the lower of those two fitted trend lines was assumed.

Assuming that there were no significant factors changing the dynamics, such a trend line should fit the observed data, whereas any divergences should be relatively small and intermingled, without any obvious pattern. In the case of any factor actually boosting the vaccination rate, it should clearly break any trend line and the divergence should roughly match the moment when the factor becomes relevant. Additionally, if the previously observed pattern [26] for Lithuania’s vaccination rate, very similar to the Polish trend, still holds, then Lithuanian values should stay within 2 standard deviations of observations for 16 Polish provinces of comparable size or be explainable by identifiable subsequent events.

Model fitting was done using a specially written Python script that relied on libraries pandas and statsmodels.

## 3. Results

**Figure 1:**
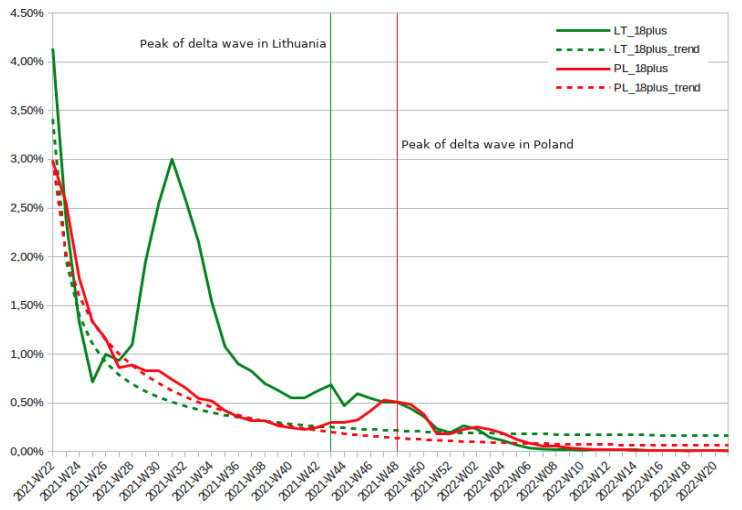
Percentage of Polish and Lithuanian adult population that received the first dose of COVID-19 vaccine in the period 2021-W22 to 2022-W21.

As presented in Figure 1, the trend line for Poland matches relatively closely the actual number of adult Poles receiving the first dose of COVID-19 vaccine in the analysed period. However, the trend line persistently underestimated the number of those vaccinated in the period 2021-W42 to 2022-W06, with the highest surplus of people taking the first dose of the vaccine a week before the number of detected delta variant cases peaked. While the estimated trend line for Lithuania is relatively similar, the actual vaccination rate in that country diverges from that trend more than once. There is a huge vaccination spike at approximately 2021-W31, when the Lithuanian parliament was proceeding with the restrictive COVID-19 passport law. Moreover, there is a smaller vaccination rate spike at approximately 2021-W44, at exactly the time when Lithuania was experiencing a peak of the delta variant wave. Besides, in both countries there is a vaccination dip near Christmas and New Year.

**Figure 2:**
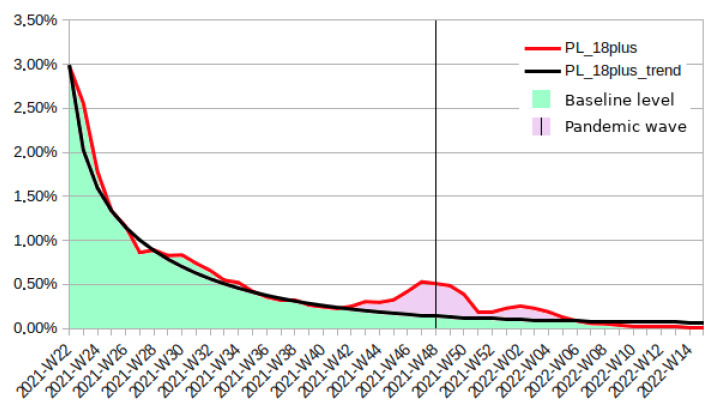
Percentage of Polish adult population that received the first dose of COVID-19 vaccine in the period 2021-W22 to 2022-W15, subdivided into groups according to different implied motivation mechanisms.

In Figure 2, the number of vaccinated Polish adults is divided according to implicit causes. Based on the trend line, 19.96% of population decided to vaccinate in almost exactly the weekly numbers matching the trend line. Nevertheless, when the number of infection cases was growing, the number of people receiving the vaccine increased clearly above the trend line and an additional 3.20% of the population received the vaccine as well.

**Figure 3:**
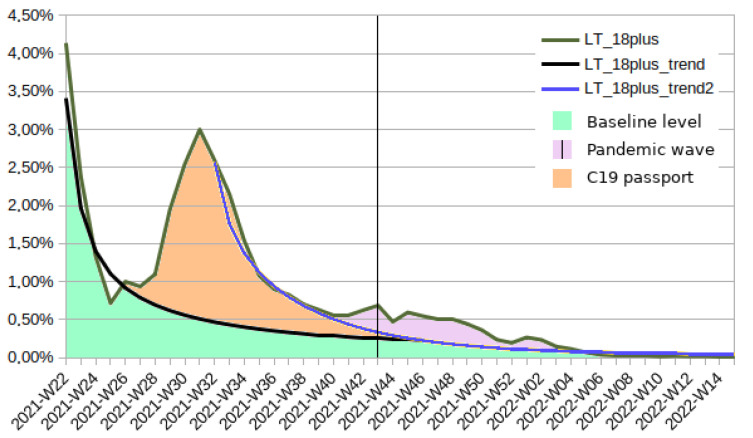
Percentage of Lithuanian adult population that received the first dose of the COVID-19 vaccine in the period 2021-W22 to 2022-W15, subdivided into groups according to different implied motivation mechanisms.

The situation in Lithuania, presented in Figure 3, is more complex than that in Poland. The trend line was fitted twice, first to match the vaccination decrease in mid-summer, second—to match the new trend line after the initial impact of the COVID-19 passport started to fade. Based on picking the lower of those two lines, the percentage of Lithuanians who simply needed more time to make the decision to get vaccinated was 19.06%. According to the model, the COVID-19 passport was responsible for convincing an additional 13.98% of the adult population. This restriction came into force on 13 September 2021 (first day of 2021-W37). Before this date, 10.86% of the compelled population received at least a single dose of the vaccine, while in the period when the passport was actually in force—an additional 3.12% followed suit. Moreover, similar to Poland, there was a vaccination surge in Lithuania when the number of infection cases increased, a fact which was responsible for the vaccination of an additional 3.89% of the adult population.

Table 1 presents disaggregated data on the percentage of the population of Polish provinces that received the first dose of COVID-19 vaccine in the period from 2021-W22 to 2022-W21. There are a few clear patterns—the younger the age group, the higher the number of people who received vaccine, though this pattern is consistent with the fact that vaccination of older age groups started earlier and thus had less time to finally make up their minds. While in younger groups there was a higher number of unvaccinated people, there is a noteworthy pattern that the lower the age group, the higher the percentage that were actually convinced to vaccinate by an infection wave. Even in relation only to the number of those remaining unvaccinated as of 2021-W21 in their age group, the share of those encouraged this way in the 18–24 age group was 1.81 times higher than among those aged 80 or more.

A somewhat similar pattern emerged for Lithuania, which is presented in Table 2. There, the outcome of the introduction of the COVID-19 passport is also visible. Nevertheless, the passport turned out to be the most effective in the lowest risk groups, i.e., the younger—it convinced as many as 19.75% of people in the 18-24 age bracket, while only 3.88% of those 80 years old or older were convinced. Even in relative terms, out of those who as of 2021-W21 remained unvaccinated, it convinced 26.10% of those aged 18–24, 26.89% in the 25–49 age group, 27.47% of those aged 50–59, 22.98% in the 60–69 group, and 16.89% in the 70–79 group, but only 8.14% of those aged 80 and more. The pattern from Poland, where the younger the person, the more convincing an argument the infection waves were, was not observed in Lithuania, though the introduction of the COVID–19 passport there might have led to a situation where the pool of young, slightly hesitant people could have been simply exhausted.

The results of paying €100 to people in the 75 or more age group are subtle but noticeable. Defining their exact starting point is challenging, as this regulation was introduced when the epidemiological situation was becoming serious but the law had been debated for months before its actual implementation. However, assuming that such a handout worked, this group should be an outlier during the delta variant wave. As presented in Table 2, when the impact of the COVID-19 passport is filtered out, Lithuania’s vaccination rate stays well within the range observed among the Polish provinces. There is only one Lithuanian value that differs by more than 2 standard deviations—its vaccination rate among people 80 years and above; in that group it diverges from the values observed in Poland by 5.36 standard deviations. Data are aggregated for the whole 70–79 year bracket, so this metric does not bring conclusive results for the 75–79 age group. Using Poland as a baseline, this implies that in the 80 or older age group as well 1.62% were convinced by an infection wave as such, while the surplus of 1.32% was actually convinced by the financial reward. Assuming the dispersion observed for Polish provinces and taking a margin of error of 2 standard deviations, the confidence interval should be ±0.5%.

However, there is also additional indirect evidence that this cash handout must have had some impact. In order to receive the second dose before the deadline, an elder had to receive the first dose by 2021-W45, and exactly at that particular moment there is a spike of vaccinations in both the 70–79 and the 80 or more age group, which—in contrast to all previous spikes caused by the introduction of the COVID-19 passport and by infection waves—is not in the least mirrored in the other age groups.

## 4. Discussion

Compared with other published works, our study is novel in several respects. To begin, it analyses actual vaccination outcomes instead of the intentions declared by respondents [36,37]. It has been reported that there may be considerable divergence between declarations and actual behavior [30,38,39,40]. The actual (i.e., based on vaccination outcomes, not on declared intentions) effectiveness of a financial reward has not been sufficiently tested worldwide since few countries decided to implement that measure and thus enable a natural social experiment, especially that the very idea raises moral doubts [41,42,43,44,45]. Limited research from Germany would suggest that relatively small monetary incentives might be effective in increasing vaccine uptake [46], whereas an American study found that small financial incentives can offset costs related to lost wages, transportation, and childcare [47]. Some other researchers also focused on the ethical side of resorting to potential financial incentives [41,45] or the idea of various types of “punishment” for the unvaccinated [48,49]. However, to the best of our knowledge, ours is the only paper that explores the hard vaccination data, i.e., concentrates on the efficiency of the said policies. As such, it may offer certain insight and feedback for national policy-makers concerned with vaccine hesitancy, which has been widely reported to be a serious problem in many countries. The strength of our findings lies also in the fact that it offers practical suggestions targeted at particular age groups, since different motivators seem to appeal to different segments of the population.

Asking about the factors potentially decisive in encouraging the population in Poland and Lithuania to vaccinate, we considered the effects of pandemic waves, of the

COVID-19 passport, and of the €100 reward for seniors. Although ¾ of Lithuanians who were convinced by the passport was vaccinated before the first person was actually asked to show it, Lithuania had higher weekly vaccination rates than Poland until 2021-W47, and even then the increase in Poland was caused by a pandemic wave. Moreover, it seems that the passport was not speeding up the decision but almost exclusively motivating those who otherwise would not have been vaccinated. Had there been a high share of the population who genuinely needed just more time, Poland’s weekly vaccination rate should have overtaken that in Lithuania early. The only period after its introduction when Poland was vaccinating weekly a higher share of adults than Lithuania was the rather late period between 2022-W02 and 2022-W10, and even there the whole surplus was a tiny 0.38%.

Paying the oldest people was also of limited efficiency. According to the model, it convinced 1.32% of people in age group 80 and above. However, this unimpressive number should be taken in perspective, as in this demographic 1.62% were convinced by a pandemic wave, 3.88%—by the COVID-19 passport, while 6.09% simply needed extra time. Confronting the number that was influenced by this incentive with the number of people in the 80 or more age group who received the first dose of the vaccine between the first day of September and three weeks before the full-vaccination deadline (2021-W35 to 2021-W45), we can see that only 29.4% of those entitled to the money were actually motivated by the scheme. What is noticeable is there was a slight surge in vaccination just before the deadline, implying that the encouraged people may have been not necessarily hesitant but just procrastinating.

The COVID-19 passport seems to be an effective tool when the goal is to achieve a high vaccination rate aimed at reaching herd immunity, although when the vaccines turn out to have a limited and short efficiency against symptomatic infection, most of this rationale is lost. Setting aside the elusiveness of achieving herd immunity, however, there is an additional problem with a heavy-handed and blunt approach. The key remaining elder population turned out to be less responsive than low-risk groups and thus any uniform policy that convinces them is likely to be excessive in more responsive younger groups.

The present 2021–2022 data seem to corroborate our earlier findings on Lithuania [26]. We found, however, that the effect of the COVID-19 passport in that country, visible directly after its coming into force (and even somewhat earlier), did not extend into 2022. Time also showed that Poland did not decide to introduce the COVID-19 passport, which enables us to compare in retrospect the vaccination rates for both countries until this day.

It is interesting to juxtapose our data against the results of a national survey conducted in Lithuania in August 2021 [50] that aimed, among others, to establish what would encourage respondents to vaccinate. The possible answers included: cash gifts, a lottery, the threat of dismissal, a huge increase in infection rates, privileges for vaccines, and evidence that vaccines are safe. It was also possible to give the option “other”—and, interestingly, that was the most popular choice among the unvaccinated respondents, chosen by more than half of them, with evidence that vaccines are safe ranking second in popularity (37%)—this reason was the most popular in the 18—44 age group. Regrettably, beyond that the survey data available in the media were not broken into age groups, so indirect information can be derived only from social status, and this fact allows a conjecture that “a huge jump in infection rates” would be most convincing to young people (represented in the survey as school and university students), and least—to the oldest respondents (OAPs in the survey). Rather surprisingly, the option of a “cash gift” was chosen by not only none of the students but also none of the OAPs, which agrees with our present findings that the effect of the €100 handout, although visible, was weak.

This is also in line with the results of another all-Lithuanian survey, commissioned by the portal LRT and conducted by Norstat on a group of 1000 adults, with the age-, sex-, and residence-specific percentages applied, in November 2021 [51]. The respondents were asked how they evaluated the opportunity passport and what most prompted them to get vaccinated against COVID-19. Interestingly, only a small proportion of respondents (6%) indicated that it was the opportunity passport that had motivated them, with an additional 6% who claimed doing so for fear of losing their jobs (which might show they were indirectly affected), while this study suggests that the passport convinced 13.98% of the Lithuanian adult population. The pattern of the answers roughly matches our vaccination data: the lower the age group, the higher the percentage of those admitting getting vaccinated for non-medical reasons. Based on the survey, the opportunity passport worked best for the two lowest age groups, 18–24 and 25–34, in which 13–14% of those surveyed declared its influence in their decision, whereas its effect was non-existent in the 75 or more age bracket. Confronting those results with our data indicates that there was some rationalisation of prior decisions on the part of the vaccinees, as our data clearly show that the COVID-19 passport did work, though weakly, even in the oldest groups. The opposite result was visible in the younger age brackets, especially among those aged 18–24, where vaccinating either because of the passport (13%) or for fear of losing a job (11%) was actually declared by a higher share than our model indicates (19.75%). While it is possible that some people genuinely felt anxious about their job regardless of restrictions, it is also possible that for high-risk people rationalisation was going towards internal factors, while for the lowest-risk people there was even rationalisation directed at external factors.

The complete lack of the declared impact of the passport in the oldest age group speaks in favour of the Lithuanian authorities’ decision to target payment for vaccination specifically at that age group, and, more importantly, relates to our present findings about the effectiveness (in terms of vaccination outcomes) of the said financial incentive. In addition, a tiny percentage mentioned the pressure from one’s nearest and dearest, which might explain the observed phenomena of late adoption, after one whole circle got vaccinated. The implicit role of social pressure fits well with prior Polish studies too, showing that predictors of late vaccination include a high initial vaccination rate, markers of social participation (voter turnout, employment rate), and a lack of support for either right-wing populism or parties even further diverging from the mainstream [52].

The officially announced success of the €100 handout might be debatable. According to official Lithuanian data, over 93,000 seniors fulfilled the conditions and thus became eligible to collect their money in December, with another 43,000 seniors due to receive the benefit in January. Thus, as of 6 January 2022, a total of approximately 136,000 COVID-19 vaccinated seniors (i.e., approximately half of those entitled, as the number of Lithuanian citizens aged 75 or more at the time was 272,000) received or were to receive the €100 reward in return either for first-time vaccination with one or two doses (depending on the type of vaccine) or for receiving a booster dose (if they had been so vaccinated already) [53]. However, in view of the above-mentioned August survey results (where the elderly seemed unmoved by a financial offer) and also considering our findings (which do not show such an impressive vaccination outcome success in the group aged 75 or more), we are inclined to believe that a considerable proportion of those who eventually collected their money were the recipients of the booster dose—so those who would otherwise mostly have been vaccinated anyway, without any financial incentive.

Moreover, our data lead us to believe that the financial incentive in Lithuania did motivate some of the 75 or older group; nevertheless, this was not reflected in the opinion poll [51] in which only 2% in this age group chose “other reason” (the only option under which the financial reward could be logically subsumed) as the option that convinced them to vaccinate, whereas as many as 83% explained their decision by the need to protect their health.

While filtering out the impact of relevant factors in Lithuania, one serious caveat should be noted. They were co-occurring, thus a person who was grudgingly willing to live without a COVID-19 passport was at the end of the year pushed by the COVID-19 passport, as well as by the surging infection wave, and was potentially tempted by the cash handout too. On the other hand, those more willing to bow to persuasion or pressure were already vaccinated and the remaining were those who were hard to convince. Nevertheless, judging from the fact that the share of Lithuanians convinced by a pandemic wave remained well within the variability observed in Poland, those factors seem to roughly cancel out.

## 5. Limitations

Ours is an observational study that concentrates on measuring which implicit motivating factors lead to vaccine uptake. Therefore, an absolute discrimination between cause and effect cannot be made. It is also beyond the scope of this work to analyse the ethical, legal, or technical ramifications. The applied methodology only allows for calculation of approximate values without error bars. Basing our study on hard data on actual vaccination timing instead of declared motivation increases credibility at the expense of understanding the actual thought processes of an individual. For example, vaccination increase ascribed to the COVID-19 passport includes individuals who had been vaccinated before the law was passed and could have been worried by the steadily escalating restrictions against the unvaccinated, not by the final law. Similarly, the surge in the vaccination rate during the wave could include not only people afraid of the illness itself but also those who feared neither the virus nor the vaccine but merely wanted to avoid more restrictive isolation rules for the unvaccinated. With the applied methodology we can estimate reasonably the role of general categories.

## 6. Conclusions

The COVID-19 passport was clearly effective at convincing the low- and moderate-risk groups to get vaccinated. In the case of aiming at vaccinating some fixed share of population to achieve herd immunity, it was desirable. However, the higher the risk of the group, the lower the efficiency of this mechanism. While the simplest explanation would be that the oldest people are least affected by this policy, as barred activities were often outside their reach anyway; however, there seems to be an additional factor. In Poland, there was a pattern that even in relative terms the youngest people were more convinced to vaccinate by a pandemic wave than the oldest. On its own, it would make no sense that the elders, who were seriously risking death, stayed unruffled, while teenagers, risking at worst a nasty influenza like-illness, panicked.

The effectiveness of the financial incentive, a measure employed in Lithuania in the 75 and older age group only was of limited success as well. However, in this high risk group there were only the relatively most stubborn holdouts left, who were very hard to convince in any way. Therefore, programmes should be better tailored to high-risk groups, if possible—skillfully combining both negative and positive stimuli at the same time.

Another finding of this study is establishing that the effects of the COVID-19 passport had already appeared at the time of the public narration about its introduction—even before the actual implementation of this measure. These effects consist in the actual increase in the number of those vaccinated and not merely in accelerating the act of vaccination for those who simply “needed more time” but were not its opponents in principle.

Finally, we noted that successive infection waves (along with the media reporting the numbers of deaths) did not have a lasting effect but only a transient impact on the weekly vaccination rates. This phenomenon was better visible in the low-risk (i.e., younger) groups.

## Figures and Tables

**Table 1:** Polish unweighted province average (±1 standard deviation) of the percentage of people, subdivided into age groups, who received the first dose of the COVID-19 vaccine in the period 2021-W22 to 2022-W21, split according to implied motivation mechanisms.

	Trend	Infection Wave
PL_18_24	30.05% (±6.14%)	5.69% (±1.36%)
PL_25_49	23.37% (±3.85%)	5.14% (±1.25%)
PL_50_59	17.42% (±2.56%)	3.62% (±0.93%)
PL_60_69	11.70% (±1.52%)	2.36% (±0.51%)
PL_70_79	7.42% (±1.25%)	1.75% (±0.32%)
PL_80plus	5.27% (±0.92%)	1.62% (±0.25%)

**Table 2:** Percentage of Lithuanians (subdivided into age groups) that received the first dose of the COVID-19 vaccine in the period 2021-W22 to 2022-W21, split according to the implied motivation mechanisms, with information in brackets by how many standard deviations this value diverges from the values observed in Poland. Note the outlier in bold.

	Trend		Infection Wave		COVID-19 Passport
LT_18_24	32.57%	[0.41 SD]	3.54%	[−1.58 SD]	19.75%
LT_25_49	25.25%	[0.49 SD]	4.99%	[−0.12 SD]	17.77%
LT_50_59	17.79%	[0.15 SD]	3.70%	[0.09 SD]	14.72%
LT_60_69	12.38%	[0.44 SD]	2.83%	[0.92 SD]	9.33%
LT_70_79	6.65%	[−0.61 SD]	2.21%	[1.44 SD]	5.47%
LT_80plus	6.09%	[0.90 SD]	2.94%	**[5.36 SD]**	3.88%

## Data Availability

Not applicable.

## Abbreviations

COVID-19: the coronavirus disease 2019, SD: standard deviation, p.p.: percentage points.
